# Telomere and subtelomere high polymorphism might contribute to the specificity of homologous recognition and pairing during meiosis in barley in the context of breeding

**DOI:** 10.1186/s12864-023-09738-y

**Published:** 2023-10-26

**Authors:** I. M. Serrano-León, P. Prieto, M. Aguilar

**Affiliations:** 1 Plant Breeding Department, Institute for Sustainable Agriculture, Agencia Estatal Consejo Superior de Investigaciones Científicas (CSIC), Avenida Menéndez Pidal S/N., Campus Alameda del Obispo, 14004 Córdoba, Spain; 2 Área de Fisiología Vegetal, Universidad de Córdoba, Campus de Rabanales, Edif. C4, 3ª Planta, Córdoba, Spain

**Keywords:** Meiosis, Chromosome dynamics, Genome organization, Barley

## Abstract

Barley (*Hordeum vulgare*) is one of the most popular cereal crops globally. Although it is a diploid species, (2*n* = 2x = 14) the study of its genome organization is necessary in the framework of plant breeding since barley is often used in crosses with other cereals like wheat to provide them with advantageous characters. We already have an extensive knowledge on different stages of the meiosis, the cell division to generate the gametes in species with sexual reproduction, such as the formation of the synaptonemal complex, recombination, and chromosome segregation. But meiosis really starts with the identification of homologous chromosomes and pairing initiation, and it is still unclear how chromosomes exactly choose a partner to appropriately pair for additional recombination and segregation. In this work we present an exhaustive molecular analysis of both telomeres and subtelomeres of barley chromosome arms 2H-L, 3H-L and 5H-L. As expected, the analysis of multiple features, including transposable elements, repeats, GC content, predicted CpG islands, recombination hotspots, G4 quadruplexes, genes and targeted sequence motifs for key DNA-binding proteins, revealed a high degree of variability both in telomeres and subtelomeres. The molecular basis for the specificity of homologous recognition and pairing occurring in the early chromosomal interactions at the start of meiosis in barley may be provided by these polymorphisms. A more relevant role of telomeres and most distal part of subtelomeres is suggested.

## Core ideas

- Barley is a useful species for wheat breeding
- Unzip the molecular bases for homologous chromosome recognition in meiosis is essential for plant breeding
- Telomeres and subtelomeres contribute to chromosome recognition and pairing during meiosis in barley
- A molecular analysis of barley subtelomeres revealed a high polymorphism for all the features analyzed

## Introduction

Barley (*Hordeum vulgare*) is one of the first domesticated plants and is also the fourth most popular cereal crop globally [83]. Barley has three primary applications: malt production, human consumption, and animal feed [59]. In a breeding context, barley is frequently employed in crosses with other cereal crops, such as wheat, to provide them with advantageous characteristics. For instance, the incorporation of both wild and cultivated barley species (*Hordeum chilense* and *H. vulgare*) into bread wheat were established several years ago [34, 62]. These methods have not only been used to transfer desirable agronomic traits into wheat but also to investigate chromosome dynamics [17, 65, 82]. Thus, breeders can develop interspecific genetic crosses to obtain new genetic combinations to be used as new crops or enlarge the genetic basis of current crops. Unfortunately, hybridization between wheat and a related species such as barley produces only a low level of chromosome pairing and recombination having undesirable implications in the transfer to wheat of alien genes controlling important agronomical traits. Therefore, understanding the genome organization and how chromosomes interact at the beginning of meiosis in both species is key for genetics and plant breeding purposes.

Chromosome recognition and pairing must occur at the beginning of meiosis. Information about other meiotic processes, such as the formation of the synaptonemal complex, recombination and chromosome segregation is available [13, 41, 43], but how chromosomes specifically identify a partner to properly pair for further recombination and segregation remains to be elucidated. Because chromosome recognition and pairing are highly dynamic processes that only occur between specific chromosome regions and may not be synchronized from one nucleus to the next, these initial chromosome interaction studies are still challenging [112]. In higher eukaryotes, telomere-mediated reorganization at the beginning of meiosis seems to be a widely conserved first step in the homology search process of homologous chromosome recognition and pairing [14, 91, 112]. A DNA conformational change has been described in wheat and barley chromosomes when telomeres (and subtelomeres) correctly associated at the onset of meiosis, which is triggered along the chromosomes and is directly correlated with homologous recognition and pairing [16, 79].

Telomeres, stretches of repetitive sequences found at the ends of chromosomes, exhibit a high degree of conservation across eukaryotic organisms, also serve the essential function of preventing various chromosome-related issues, including end-to-end fusion, recombination, and the degradation of chromosome ends [49, 55–57, 60]. Moreover, these structures may also play a role in regulatory processes and meiosis [11, 12, 76, 97–99]. At the beginning of meiosis, a structure (known as “bouquet”) formed by the association and clustering of telomeres at the inner nuclear envelope in many species including wheat and barley, facilitates the initial interactions between homologues for recognition and pairing to enable subsequent recombination [8, 69, 72, 91, 113].

Additional chromosomal regions, particularly subtelomeres located adjacent to telomeres, are also considered when determining pairing specificity as chromosomes come into close proximity within the bouquet structure. This is because the telomeric DNA sequence itself is highly conserved [16]. Although subtelomeres are an attractive target for research, their polymorphism presents a technical obstacle [1]. Subtelomeres play a role in telomere maintenance through processes such as recombination or epigenetic modification. They are gene-rich areas, but they are less conserved than telomeres and often contain recombination hotspots [86, 21, 47, 51]. Furthermore, it's worth noting that the nucleotide sequences of telomeres and subtelomeres in many sequenced genomes have not been comprehensively characterized, despite their significant functional relevance [45, 57, 63], making the evaluation of their putative conserved roles more difficult.

The majority of studies on subtelomeres have concentrated on the distal 500 Kb of each chromosome arm in the species under investigation, including humans, *Arabidopsis*, rice, and wheat [1, 40, 52, 53, 63]. However, the detailed molecular organization of subtelomeres is still unclear.

Several hypotheses regarding the functions of subtelomeres in chromosome dynamics and genome stability have been proposed due to their high polymorphism. For instance, in rice, subtelomeres may play a role in promoting recombination and the insertion of transposons [23]. In wheat and rye, recombining areas are frequently involved in chromosome recognition and pairing between homologues [101]. In fact, in all eukaryote kingdoms, including plants, recombination occurs significantly in the subtelomeric region [26, 47, 51, 89]. The presence of an extra pair of barley homologous chromosomes with terminal deletions in the wheat background has further highlighted the significance of subtelomeres in the processes of chromosome pairing and recognition. This is evident as chromosome recognition, pairing, and recombination fail when subtelomeres are absent [16].

Therefore, the subsequent crucial step involves conducting an in-depth molecular analysis of both telomeres and subtelomeres to gain a deeper understanding of their structural characteristics and any distinctive traits that may be associated with the initial stages of chromosome recognition and pairing. A comprehensive analysis has previously been carried out in wheat, revealing substantial polymorphism among homologous chromosomes across all the examined features [1].

In this study, we have identified and characterized the telomeric and subtelomeric sequences in barley chromosome arms where telomeric sequences were annotated (2H-L, 3H-L, and 5H-L), thereby enabling the precise delineation of the subtelomeric sequence's starting point. To the best of our knowledge, this represents the first molecular analysis of barley telomere sequences to date. Additionally, we have undertaken a detailed examination of subtelomeres within the 500 Kb distal subtelomeric regions and expanded this investigation to cover a broader chromosome region (5 Mb) in those chromosomes where telomeric sequences were identified. Our findings contribute to our understanding of the molecular structure of barley telomeres and subtelomeres and raise the possibility that the distinct patterns of various DNA repetitive sequences and DNA protein-binding sequences may play a role in chromosome specificity, which is required for homologous chromosome associations and recombination.

## Materials and methods

### Plant material and fluorescence in situ hybridization

Barley root tips from *H. vulgare* were used for in situ hybridization experiments. Three DNA probes were fluorescently labelled following standard procedures, the barley subtelomeric sequence HvT01 [10], the highly conserved telomeric sequence pAt74 originally isolated from *A. thaliana* [85], and the GAA satellite sequence isolated from barley [74] for identifying chromosomes. All the methods for preparing mitotic chromosomes spreads and in situ hybridization experiments have been described previously [77, 78].

### DNA sequences

All the sequences analyzed in this study were obtained from NCBI (RefSeq: MorexV3 https://www.ncbi.nlm.nih.gov/assembly/GCF_904849725.1).

### DNA sequence analysis and prediction tools

Telomere analysis was done utilizing NCBI sequences. Plots of this analysis were done with the informatic software GraphPad Prism 6.

Subtelomere analysis was done as follows. Prediction of coding genes and non-coding RNAs was done using *EnsemblPlants* (https://plants.ensembl.org/index.html). In *EnsemblPlants*, coding genes were detected by IPK database (Leibniz Institute of Plant Genetics and Crop Plant Research) and non-coding RNAs were detected by EoRNA database (Barley Expression Database that displays gene and transcript abundance using Barley Reference Transcript (BaRTv1.0) from The James Hutton Institute).

Low complexity domains (A, AG and G rich domains), simple repeats, DNA transposons, long terminal repeats (LTR), type I LINE transposons and type I SINE transposons were detected using *RepeatMasker* (Interspersed Repeat Masking Based on Protein Similarity, http://repeatmasker.org/cgi-bin/RepeatProteinMaskRequest) and Censor (https://www.girinst.org/censor/index.php). Parameters for the analysis with Censor were sets as follows: the sequence source used was Triticum, and this source was composed of wheat, barley and rye.

Emboss CpG plot (https://www.bioinformatics.nl/cgi-bin/emboss/cpgplot) was used for CG content calculation and CpG island prediction [84]. Parameters for the analysis were as follows: window Size (100), minimum length of a reported island (200), minimum observed/expected before a CpG island is reported (0.6), minimum average percentage of C plus G in a set of 10 windows that are required before a CpG island is reported (50).

Hot and cold recombination spots were predicted with iRSpot-EL (http://bliulab.net/iRSpot-EL/) [50]. Size of sliding window (in Kb) and step size parameters were set at 2 and 200, respectively.

Sequences associated with hot recombination spots [19] were identified (simple repeat: CCGCCGCCG, and sequences associated with transposable elements: CTCCCTCC, TTAGTCCCGGTT). These sequences were localized and displayed by means of MAST (MEME Suite 5.0.5) (https://meme-suite.org/meme/tools/mast) [7]. Parameters were set as follows: direct and reverse complement sequences were analyzed, and results combined, E-value ≤ 10 (MAST displays all sequences, exact or degenerate, matching query with E-values below the given specified threshold), the *p*-value of a hit must be less than 0.0001 to be shown in the output.

Distribution of predicted DNA-binding sites of putative barley proteins homologous to SMC1β cohesin (CCACCAGGTGGC), YY1 (GGGGGCAGTGG) and HMG proteins ([AT] *n* > 5) was obtained by means of MAST (MEME Suite 5.0.5) (https://meme-suite.org/meme/tools/mast) [7]. Parameters were set as follows: direct and reverse complement sequences were analyzed and results combined, E-value ≤ 10 (MAST displays all sequences, exact or degenerate, matching query with E-values below the given specified threshold); the *p*-value of a hit must be less than 0.0001 to be shown in the output.

In both regions (telomere and subtelomere), DNA analyser (G4 hunter) (https://bioinformatics.ibp.cz/#/) has been used for the study of G-quadruplexes. For this analysis we tried the standard threshold (1.2), but we finally used a more restrictive threshold (1.8).

## Results

**Fig. 1:**
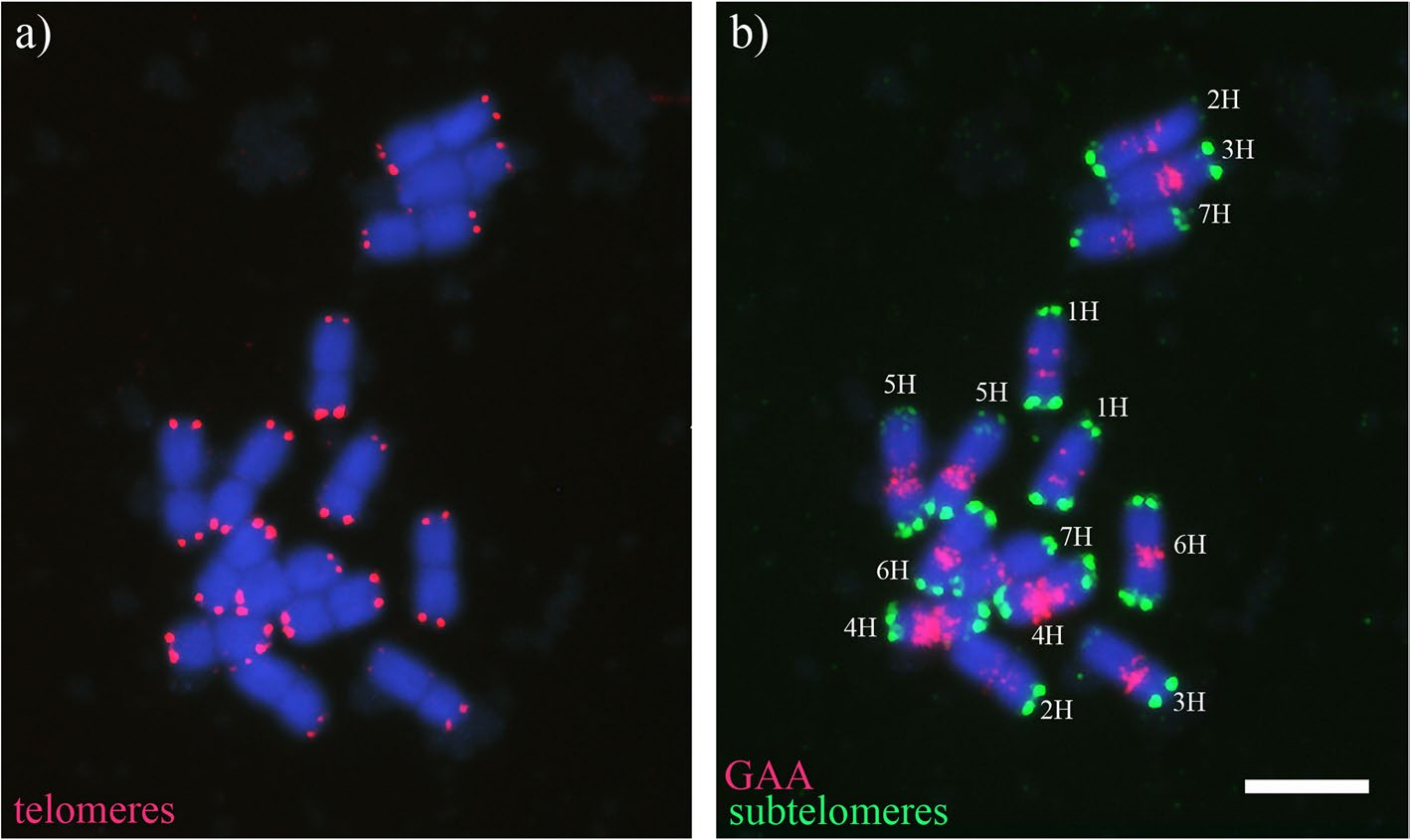
In situ hybridization (FISH) of barley chromosomes. **a** Telomeres in barley chromosomes (red). **b** Subtelomeres in barley chromosomes (green) and GAA (red). Chromosomes were counterstained with DAPI (blue). Scale bar represents 10 μm for both panels

The sequences located at the ends of all barley (*Hordeum vulgare*) chromosomes were scrutinized using the data available from NCBI. Barley has 7 pairs of chromosomes (chromosomes 1H, 2H, 3H, 4H, 5H and 7H). In situ hybridization experiments were performed in somatic metaphase chromosome spreads to visualize the telomeric repeat and the subtelomeric region on barley. As expected, all barley chromosome ends contained the telomeric repeat and a high variability for the subtelomeric sequence was found among different barley chromosomes (Fig. 1). However, we found that only the assemblies of chromosomes 2H, 3H and 5H long arms contained plant terminal telomeric repeats (5’-TTTAGGG-3’). For this reason, we decided to restrict our study to the long arm ends of chromosomes 2H, 3H and 5H.

We focused our study on the chromosome ends comprising the telomeric repeat sequences and the distal part of subtelomeres adjacent to the telomeric repeat sequences.We identified chromosome arms that included the telomeric repeat sequence (Table 1). We found differences on the length of the three telomeres analyzed, being 3H-L chromosome telomere the longest and 2H-L chromosome telomere the shortest.

**Table 1:**
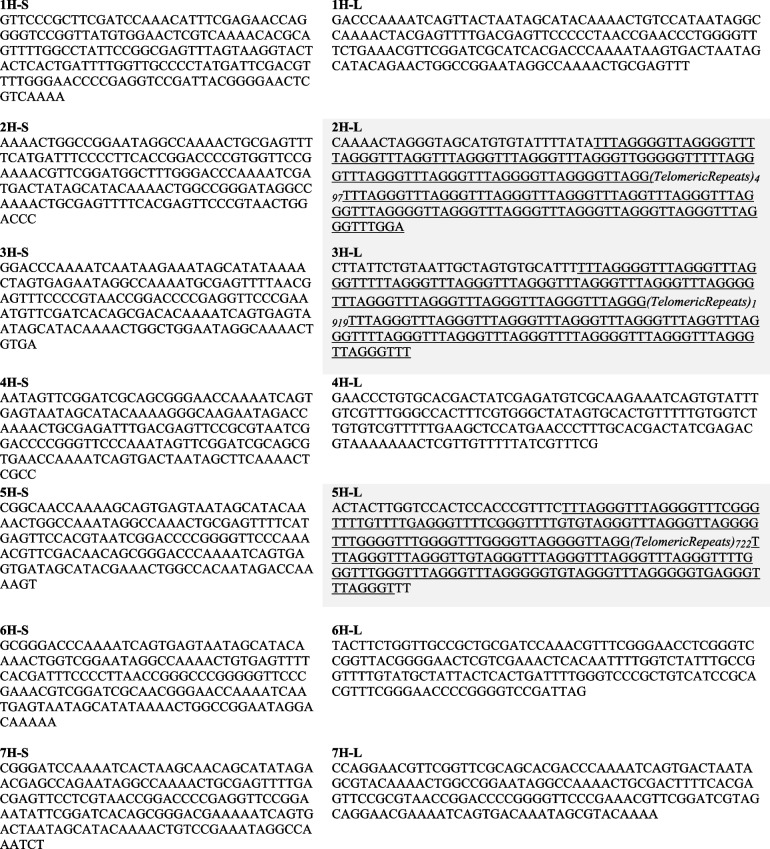
Sequences of barley (*Hordeum vulgare*) chromosome ends. All 14 chromosomes ends are displayed, including both short and long arms of chromosomes. All sequences are presented on the direction of the sequencing, from the end of the short chromosome arm to the end of the long chromosome arm. Chromosome arms that present telomeric repeats are highlighted in grey. The telomeric sequence of 2H-L, 3H-L and 5H-L chromosome arms is underlined. Sequences were obtained by ENA from EBI (RefSeq MorexV3)

**Fig. 2:**
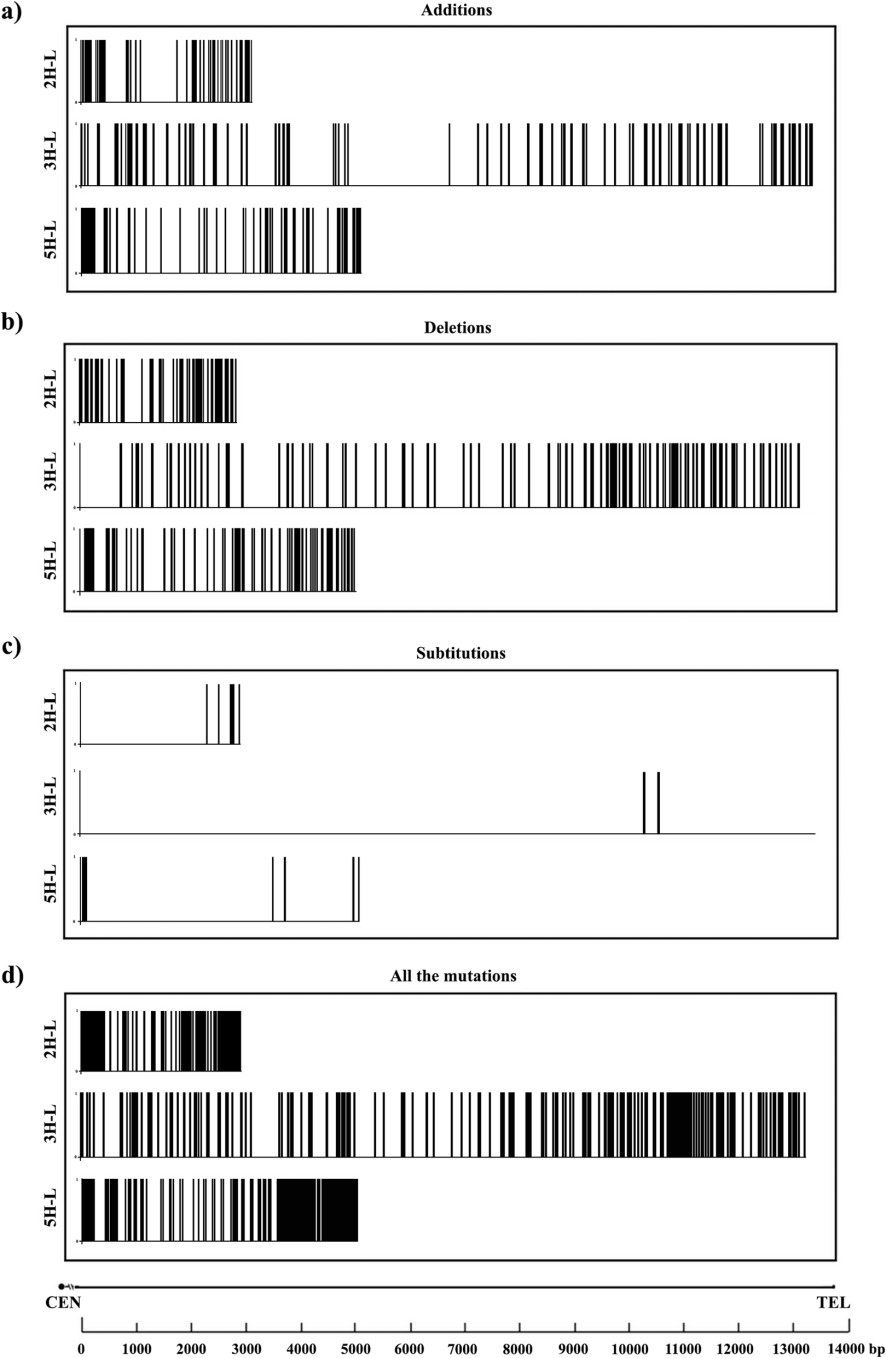
DNA variations for the repeat plant telomeric sequence (TTTAGGG) including nucleotides additions, deletions and substitutions along the telomeric sequence identified in chromosome arms 2H-L, 3H-L and 5H-L. **a** Additions (**b**) Deletions (**c**) Substitutions (**d**) All types of base mutations

By sequence analysis, we have detected nucleotide additions, deletions and substitutions within the consensus sequence that conforms this repetition unit along the telomeric sequence (Fig. 2). A small number of nucleotide substitutions have been identified in this study. We only detected a fraction of 0.16%, 0.014% and 0.13% of nucleotides substitutions in barley chromosome arms 2H-L, 3H-L and 5H-L, respectively (Fig. 2c). All of them were single base substitutions. We found that adenine was the base that suffered the most this type of mutation (50% of total substitutions). In contrast with nucleotide substitutions, a higher number of nucleotide additions and deletions were detected in this chromosome region. In detail, the percentage of additions was 1.73% on 2H-L chromosome arm, 0.81% on 3H-L chromosome arm and 1.2% on 5H-L chromosome arm. Besides, we annotated a 2.81%, 0.89% and 1.62% deletions on 2H-L, 3H-L and 5H-L chromosome arms, correspondingly (Table 2). We found additions and deletions within the whole telomeric region, reporting a higher concentration of both types of mutation on the two ends of the telomere (Fig. 2a, b). It is worthy to say that 2H-L chromosome arm contained the highest accumulation of mutations (substitutions, additions and deletions), 4.71%, in contrast with 3H-L and 5H-L chromosome arms, which only have 1.72 and 3.03%, respectively (Fig. 2d).

**Table 2:** Percentage of substitutions, additions and deletions in the telomeric sequence of 2H-L, 3H-L and 5H-L barley chromosomes

	**Substitutions**	**Additions**	**Deletions**	**All mutations**
2H-L	0.16%	1.73%	2.81%	4.7%
3H-L	0.014%	0.81%	0.89%	1.714%
4H-L	0.13%	1.2%	1.62%	2.95%

In details, numerous deletions of one of the Ts in TTT were found on the three chromosome arms that were examined, a lower number of G deletion in GGG was observed. Many additions of G were also observed in GGG. On chromosome arm 2H-L, 114 deletions were detected, of which 82% were T deletions that variated the sequence from TTTAGGG to TTAGGG. Also, we found 62 additions, 87% of these mutations were G additions, transforming the canonical repeat sequence to TTTAGGGG. 131 deletions and 115 additions were located on chromosome arm 3H-L. T deletions correspond to 88% and G additions to 76.52%. On chromosome arm 5H-L, we found 96 deletions of whom 63.54% were T deletions. On this chromosome arm, we also found 68 additions, were G additions accounted for 75% of total additions. We found substitutions too, but this type of mutation is not as common as deletions and additions on 2H-L, 3H-L and 5H-L barley chromosome arms.

**Fig. 3:**
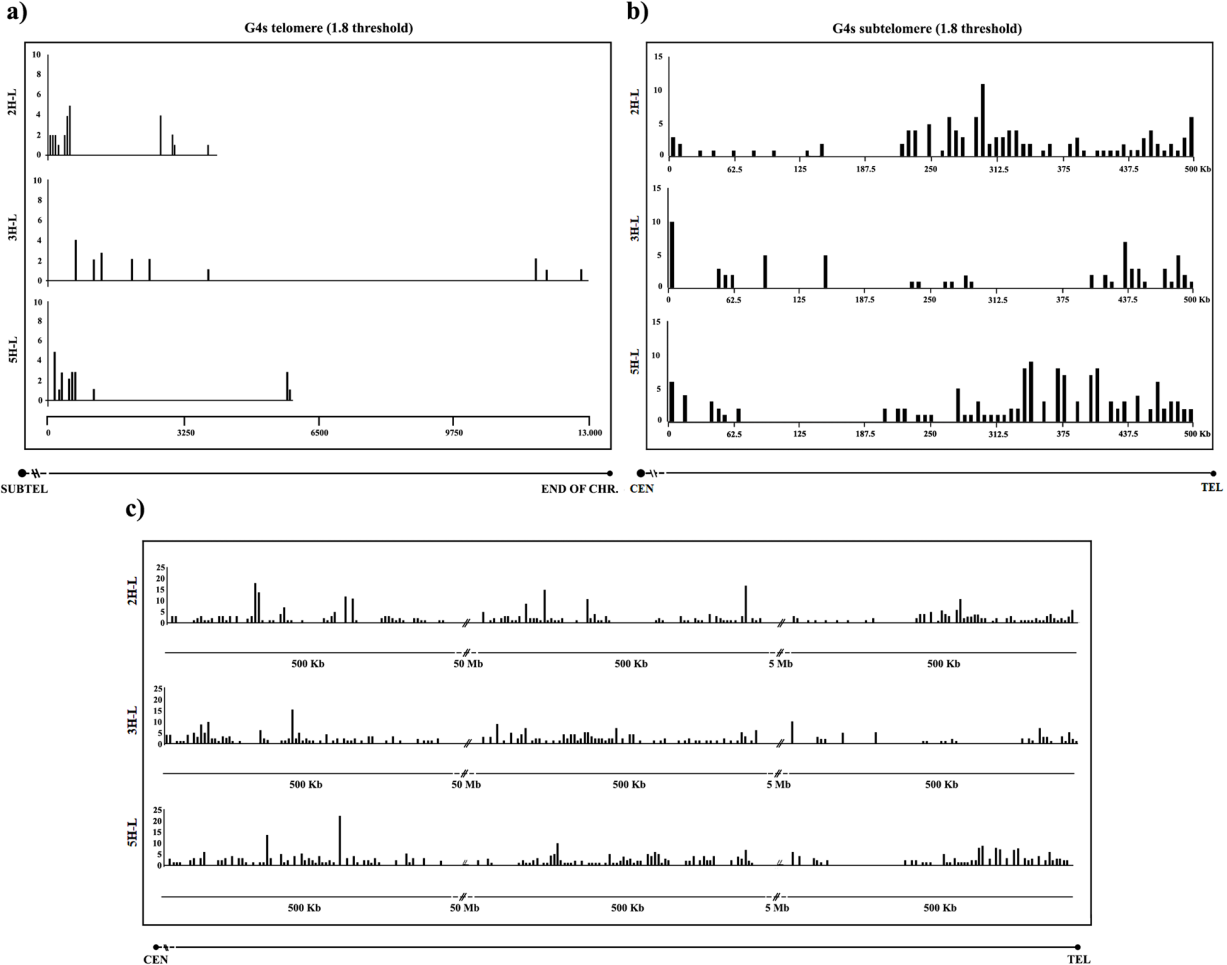
Localization of quadruplexes (G4s) within the terminal sequence of 2H-L, 3H-L and 5H-L barley chromosome arms using a threshold of 1.8 in (**a**) telomeric sequence, (**b**) subtelomeric sequence, and (**c**) three different chromosome regions from the telomeric sequence

G-Quadruplexes (G4), structures rich in guanine, were also studied in the telomere. Very different densities of G4s were found. 2H-L was the chromosome arm that presented a higher frequency of G4s (6.9/1000 bp) in comparison with 3H-L and 5H-L, which presented 1,7/1000 bp and 4.2/1000 bp, respectively (Table 3). A higher number of G4s were detected within the most distal and the most proximal parts of telomeres in all chromosome arms 2H-L, 3H-L and 5H-L (Fig. 3).

**Table 3:** Number and frequency of G4 quadruplexes identified in the telomeric sequence of 2H-L, 3H-L and 5H-L barley chromosome arms

Chromosome	Nº of quadruplexes	Frequency
2H-L	25	6.9/1000 bp
3H-L	23	1.7/1000 bp
5H-L	22	4.2/1000 bp

A characterization of the distal subtelomeric region (500 Kb) has been carried out in barley chromosome arms 2H-L, 3H-L and 5H-L, focusing on different features that could be related to chromosome recognition and pairing within the subtelomeric region: genes, transposable elements, repeat sequences, CG content and CpG island, distribution of binding sites of proteins putatively involved in chromosome pairing and recombination, as well as predicted hot/cold recombination spots. For some of the most relevant features, the analysis was extended to a larger 5 Mb sequence.

We also analyzed the abundance and distribution of G-Quadruplexes (G4) within the 500-kb distal subtelomere region of chromosome arm 2H-L, 3H-L and 5H-L. A higher concentration of G4s was found near the start of the telomere, and different abundances and distributions were found among the three different chromosome arms examined (Fig. 3). Chromosome arm 3H-L was the one that presented less frequency of G4s in its subtelomeric sequence, 0.27/1000 bp. Chromosome arm 5H-L showed the higher frequency, 0.30/1000 bp, and chromosome arm 2H-L presented a frequency of 0.28/1000 bp (Table 4). The analysis was also done on 500-kb stretches located 5 Mb and 50 Mb from the telomere, and this analysis revealed a significantly lower abundance of G4s within the 500-kb distal subtelomere in comparison with the regions closer to the centromere (Table 4, Fig. 3).

**Table 4:**
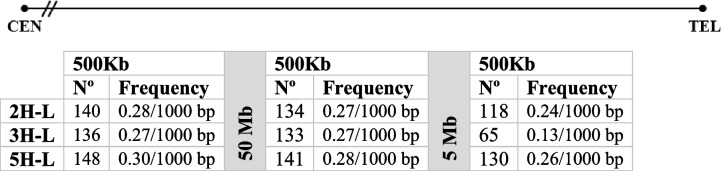
Number and frequency of G4 quadruplexes included in three regions of the distal of 2H-L, 3H-L and 5H-L chromosome arm sequences: 500 Kb of subtelomeric sequence, adjacent to the telomere, 500 Kb of a region separated by 5 Mb from the first base of the telomere and 500 Kb of a region separated by 50 Mb from the first base of the telomere

**Fig. 4:**
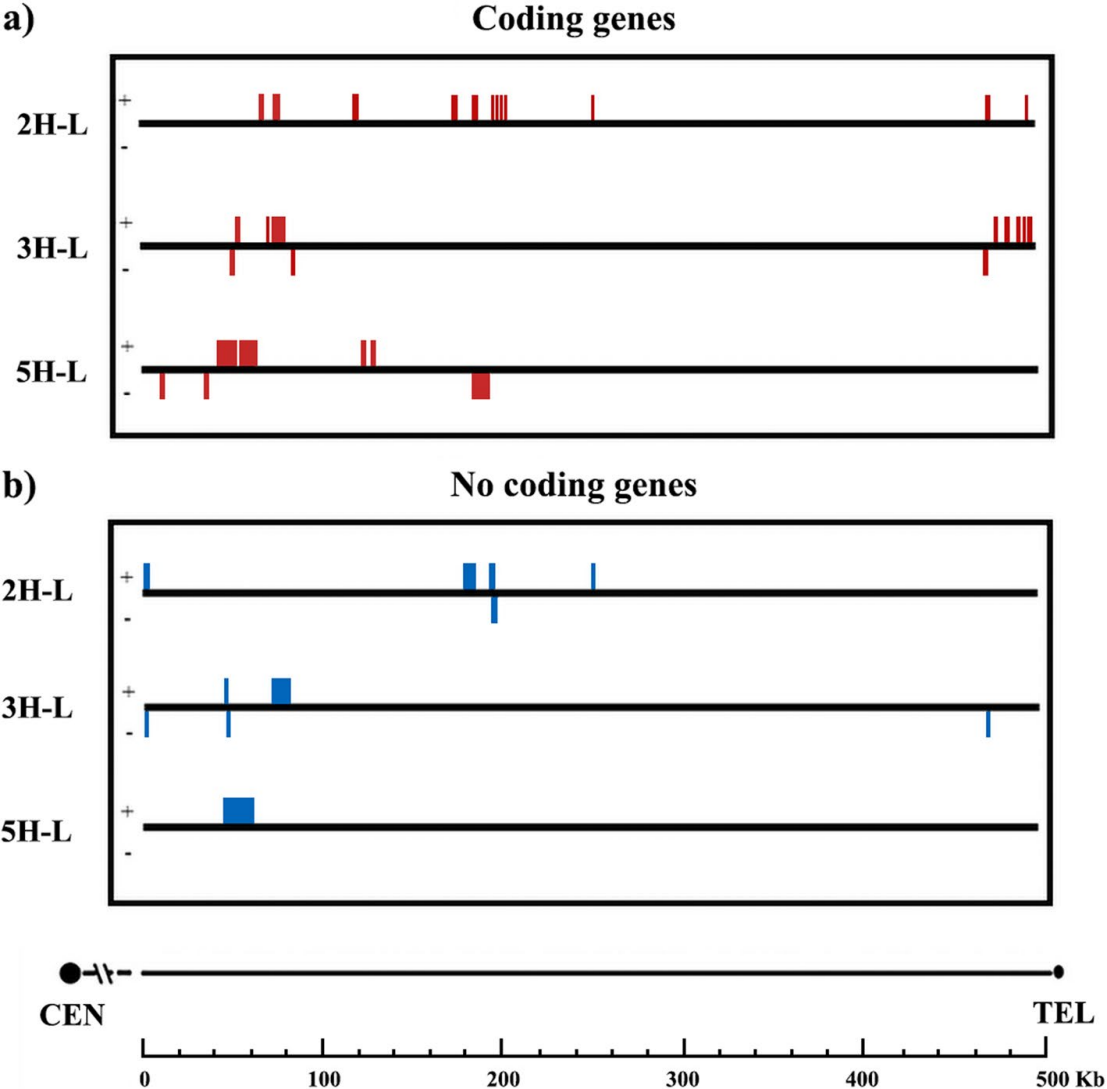
Localization of the genes included in distal subtelomeric region (500 Kb) of barley chromosome arms 2H-L, 3H-L and 5H-L. Direct and reverse complementary sequences were considered. **a** Distribution of coding genes on the three barley chromosomes, detected by IPK database (Leibniz Institute of Plant Genetics and Crop Plant Research) using *Ensembl Plants* (https://plants.ensembl.org/index.html). **b** Distribution of non-coding genes detected by EoRNA database (Barley Expression Database that displays gene and transcript abundance using Barley Reference Transcript (BaRTv1.0) from The James Hutton Institute) using *Ensembl plants* (https://plants.ensembl.org/index.html)

The location of gene sequences was also studied in barley subtelomeres (Fig. 4a: protein coding genes; Fig. 4b: RNA genes; direct and reverse complementary sequences were considered in both cases) within the distal subtelomeres of chromosomes 2, 3 and 5 long arms. A total of 43 genes were predicted in these three regions altogether within the 500 kb distal subtelomere, 30 were coding genes and 13 non-coding genes. A total of 16 genes were predicted in 2H-L barley chromosome arm, 12 of these genes were coding genes and 4 were non-coding genes. On 3H-L chromosome arm we found 18 genes, 11 were coding genes and 7 non-coding genes. Further, 9 genes were located on 5H barley chromosome arm, 7 coding genes and 2 non-coding genes. Gene density varies among the three barley chromosome ends studied. The analysis was extended to a larger region, and we identified genes within the 5 Mb distal subtelomeric sequence. In this larger region we predicted 946 genes, 255 genes in 2H-L chromosome arm, 333 in 3H-L chromosome arm and 358 in 5H-L chromosome arm (Table 5). A distinct and unique gene distribution pattern was observed across the three analyzed chromosome arms. A greater density of genes was identified in the most proximal region of the subtelomeric sequence in all three barley chromosome arms examined in this study (see Fig. 1). Conversely, the farthest portion of the subtelomere (adjacent to the telomeric sequence) exhibited a reduced gene count.

**Table 5:** Number of genes identified in 500 kb and 5 Mb of the distal subtelomeric region of barley chromosome arms 2H-L, 3H-L and 5H-L

Chromosome arm	Nº genes (in distal 500 Kb)	Nº genes (in distal 5 Mb)	Gene density (in distal 500 Kb)	Gene density (in distal 5 Mb)
2H-L	16	255	1 per 31.3 Kb	1 per 19.6 Kb
3H-L	18	333	1 per 27.8 Kb	1 per 15.0 Kb
5H-L	9	358	1 per 55.5 Kb	1 per 14.0 Kb

**Fig. 5:**
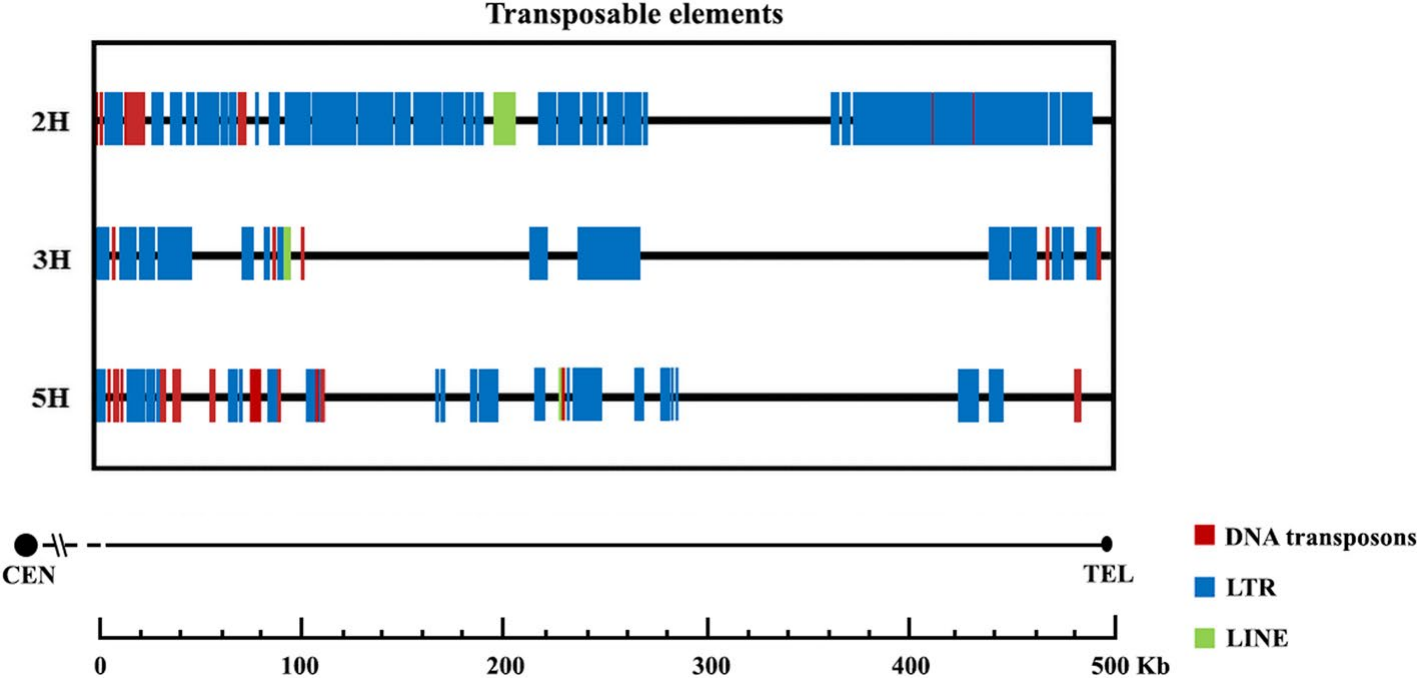
Distribution of transposable elements in barley chromosomes included within the 500 Kb distal subtelomeric region adjacent to telomeric repeats of chromosome arms 2H-L, 3H-L and 5H-L. No SINEs retrotransposons were detected in these barley subtelomeric sequences

We also analyzed the distal subtelomeric region of 2H-L, 3H-L and 5H-L barley chromosome arms, searching for the presence and distribution of TEs (transposable elements), including both retrotransposons (SINE, LINE and LTR elements) and DNA transposable elements (Fig. 5). 2H-L chromosome arm contained a high percentage of transposable elements in the 500 Kb distal subtelomere region (56.74%) compared to 3H-L chromosome arm, which only presented a 18.29% fraction of transposable elements. 5H-L chromosome arm has an intermediate percentage (36.1%). As a relevant differential attribute, transposable elements distribution pattern is chromosome specific (Fig. 5). LTR elements were analyzed. 2H-L chromosome arm presented a higher percent of LTR (52.22%), in contrast with 3H-L (17.41%) and 5H-L (31.69%) chromosome arms (Table 6). It is important to remark that only 2H-L and 5H-L chromosome arms present LINEs in a small proportion (1.73 and 0.0232% respectively), while 3H-L chromosome arm doesn’t present this type of transposable element. SINEs have not been detected on this study in any of these chromosomes (Table 6). We also studied DNA transposons. These transposable elements have been detected in greater number on 5H-L chromosome arm, taking a 4.39% of the 500 Kb sequence (Table 6).

**Table 6:** Transposable elements identified in the distal 500 Kb sequence of barley chromosome arms 2H-L, 3H-L and 5H-L

	Element	Number	Length (bp)	%
2H-L	Retroelements	715	269,739	53.95
	SINEs	-	-	-
	LINEs	17	8647	1.73
	LTR elements	349	261,092	52.22
	Ty1/*Copia*	170	108,169	21.63
	*Gypsy*/DIRS1	179	152,923	30.58
	DNA transposons	16	13,952	2.79
	Total	731	283,691	56.74
3H-L	Retroelements	77	87,052	17.41
	SINEs	-	-	-
	LINEs	-	-	-
	LTR elements	77	87,052	17.41
	Ty1/*Copia*	44	53,374	10.67
	*Gypsy*/DIRS1	33	33,678	6.74
	DNA transposons	5	4404	0.88
	Total	82	91,456	18.29
5H-L	Retroelements	355	158,621	31,71
	SINEs	-	-	-
	LINEs	1	116	0.0232
	LTR elements	177	158,505	31.69
	Ty1/*Copia*	90	85,623	17.12
	*Gypsy*/DIRS1	87	72,882	14.57
	DNA transposons	48	21,969	4.39
	Total	403	180,590	36.1

**Fig. 6:**
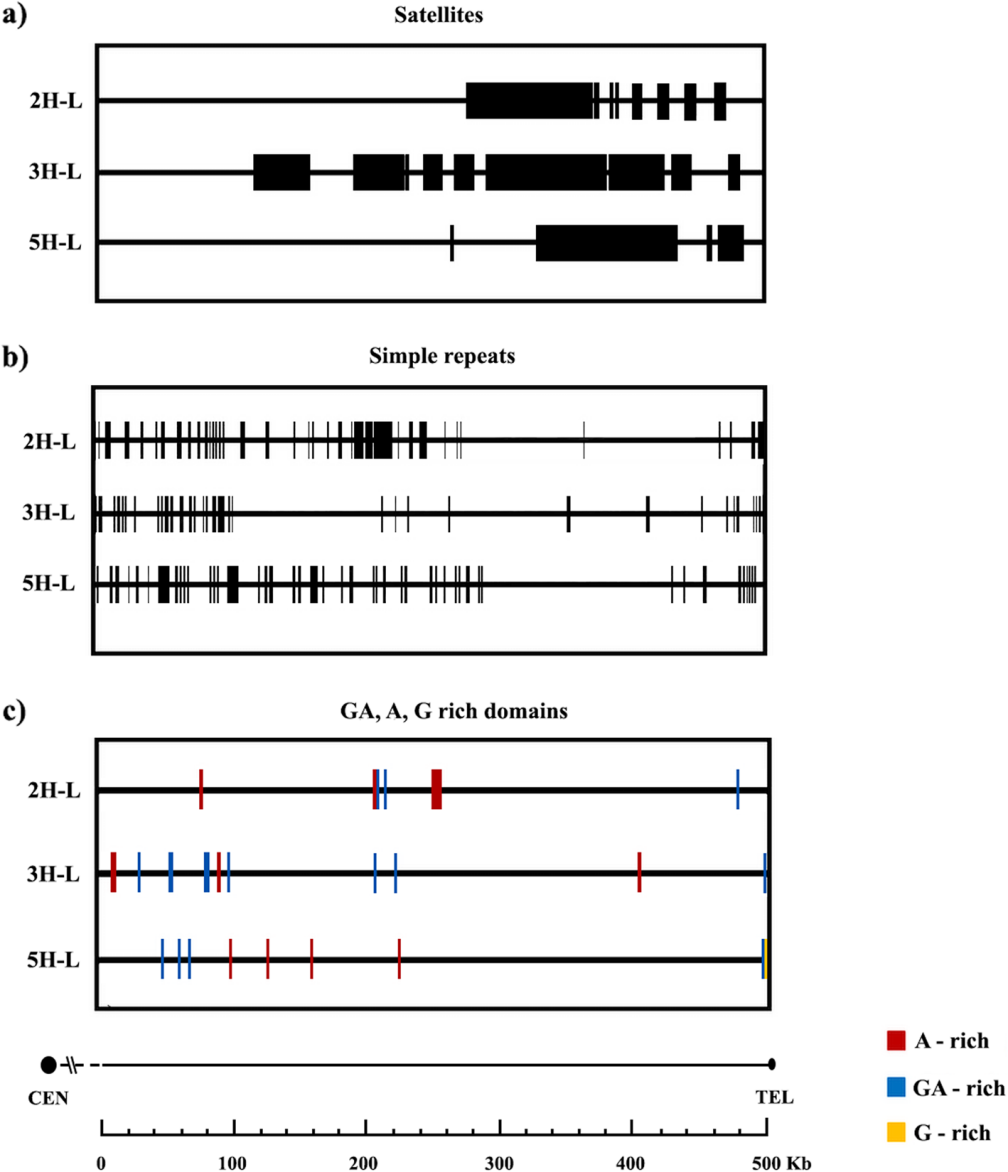
Distribution of repeat elements in barley chromosomes included within the distal (500 Kb) subtelomeric region adjacent to the telomeric repeats of chromosome arms 2H-L, 3H-L and 5H-L. (**a**) satellites, (**b**) simple repeats, (**c**) GA, A, G rich domains

**Fig. 7:**
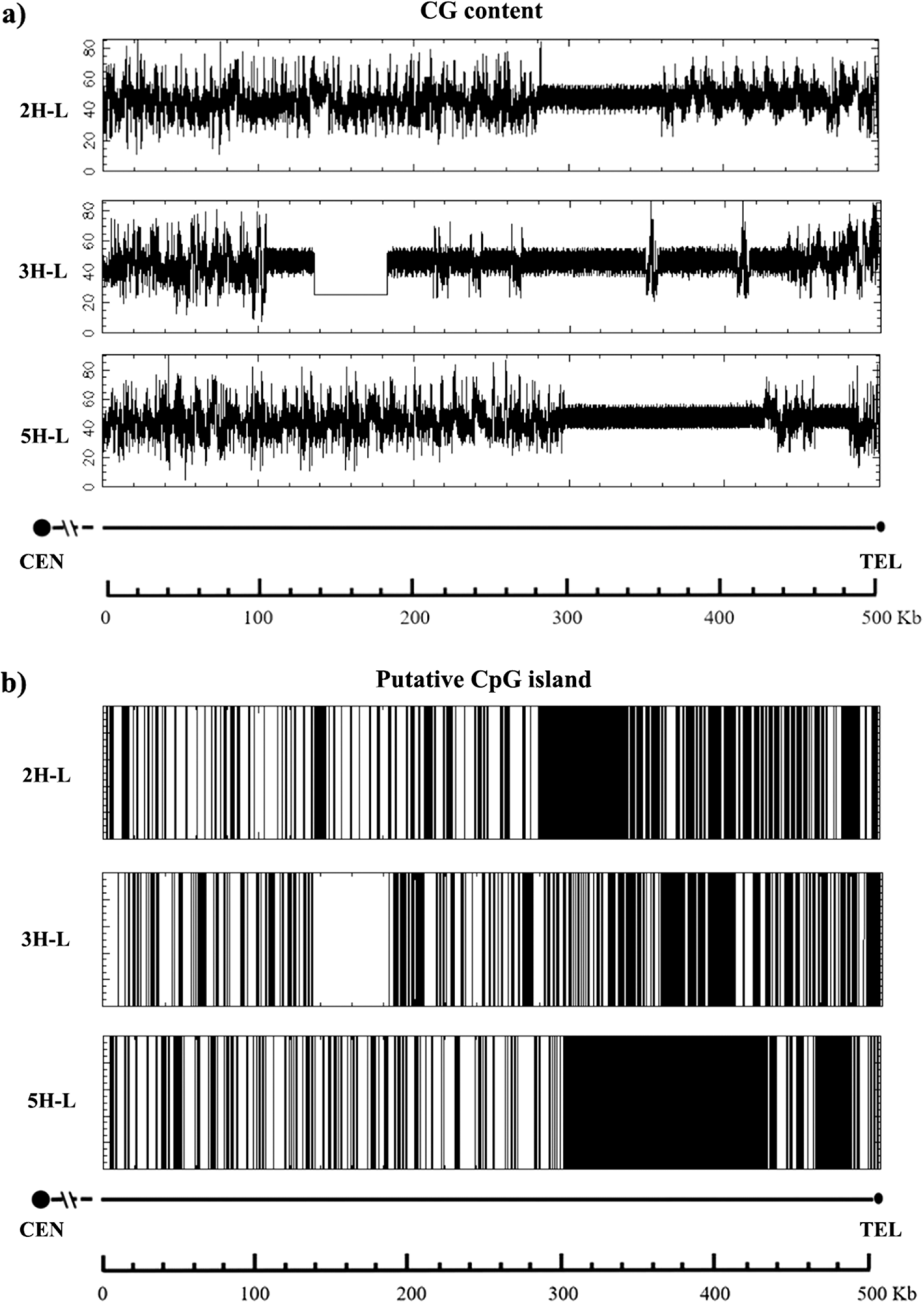
CG content and predicted CpG islands included identified in the distal subtelomeric sequence (500 Kb) of chromosome arms 2H-L, 3H-L and 5H-L. Emboss CpG plot was used for CG content calculation (**a**) and CpG island prediction (**b**). Predicted CpG islands are represented in black

The study investigated the presence and distribution of DNA repeats within the distal subtelomeric region spanning 500 Kb on barley chromosome arms 2H-L, 3H-L, and 5H-L. A quantitative analysis of these DNA repeats is shown in Table 7. Satellite repeats, consisting of multiple copies of the same DNA sequence with varying lengths, ranging from a single base to several thousand bases, were examined. Our findings indicate that these satellite repeats are primarily clustered near the telomere, especially on chromosome arms 2H-L and 5H-L. In contrast, chromosome arm 3H-L exhibited dense blocks of satellite repeats, distinguishing it from chromosome arms 2H-L and 5H-L (Fig. 6). In contrast, simple repeats, characterized by duplications of short DNA nucleotide sequences (2–5 bp), such as A, CA, CGG, and so on, display a distinct distribution pattern. Within chromosome arms 2H-L, 3H-L, and 5H-L, these simple repeats are predominantly found in the proximal region of the subtelomeric sequence (Fig. 6). Poly-purine or poly-pyrimidine stretches and regions of extremely high AT or CG content, also known as low complexity regions, were detected in all three barley chromosome arms (Fig. 6). Indeed, we noted a substantial prevalence of these repeats within the distal region of the subtelomeric sequence, in close proximity to the telomere. These low complexity regions encompass areas enriched with adenine, adenine-guanine, and guanine sequences. The occurrence of these bases within these low complexity regions exceeds 85%. Distal subtelomere sequence (500 Kb) of barley chromosome arms 2H-L, 3H-L and 5H-L long arms were analyzed for the GC content and the identification of predicted CpG islands (Fig. 7). The CG content and predicted CpG islands showed uniformity across all chromosomes. Notably, a dense concentration of CG content was observed throughout the entire set of chromosomes (see Fig. 7a, b). It's noteworthy that specific regions, located in the most distal portions of 2H-L and 5H-L chromosome arms, exhibited an approximate CG content of 50%. 3H-L chromosome arm presented also these 50% CG zones, but in this case, these zones were extended throughout the whole region analyzed. CpG islands have been observed within the whole sequence of 2H-L, 3H-L and 5H-L chromosome arm ends (Fig. 7a, b). Furthermore, we identified substantial, contiguous clusters of CpG islands in close proximity to the telomere in all three instances. These clusters coincide with satellite regions and encompass roughly 50% of the sequence (refer to Fig. 7a, b). It's worth noting that chromosome arm 3H-L contains an unsequenced stretch spanning 50 Kb, from positions 130 Kb to 180 Kb, rendering it ineligible for analysis. Moreover, none of the examined barley chromosome arms displayed noteworthy features like a high density of CG-rich DNA segments or CpG islands.

**Table 7:** Repeats elements included in the 500 Kb distal subtelomere region adjacent to telomeric repeats of chromosomes arms 2H-L, 3H-L and 5H-L. Satellites: multiple copies of the same DNA sequence, the repeated pattern can vary in length for a single base to several thousand bases long; Simple repeats: duplications of simple sets of DNA bases (2–5 bp) such as A, CA, CGG etc.; Low complexity: Poly-purine or poly-pyrimidine stretches and regions of extremely high AT or CG content

	Repeat element	Number	Length (bp)	%
2H-L	Satellites	488	114,775	22.95
	Simple repeats	103	4517	0.90
	Low complexity	17	1178	0.24
	Total	608	120,470	24.09
3H-L	Satellites	1147	256,910	51.38
	Simple repeats	80	3616	0.72
	Low complexity	14	752	0.15
	Total	1268	261,278	52.19
5H-L	Satellites	634	142,235	28.44
	Simple repeats	118	6668	1.33
	Low complexity	10	415	0.083
	Total	762	149,318	29.85

**Fig. 8:**
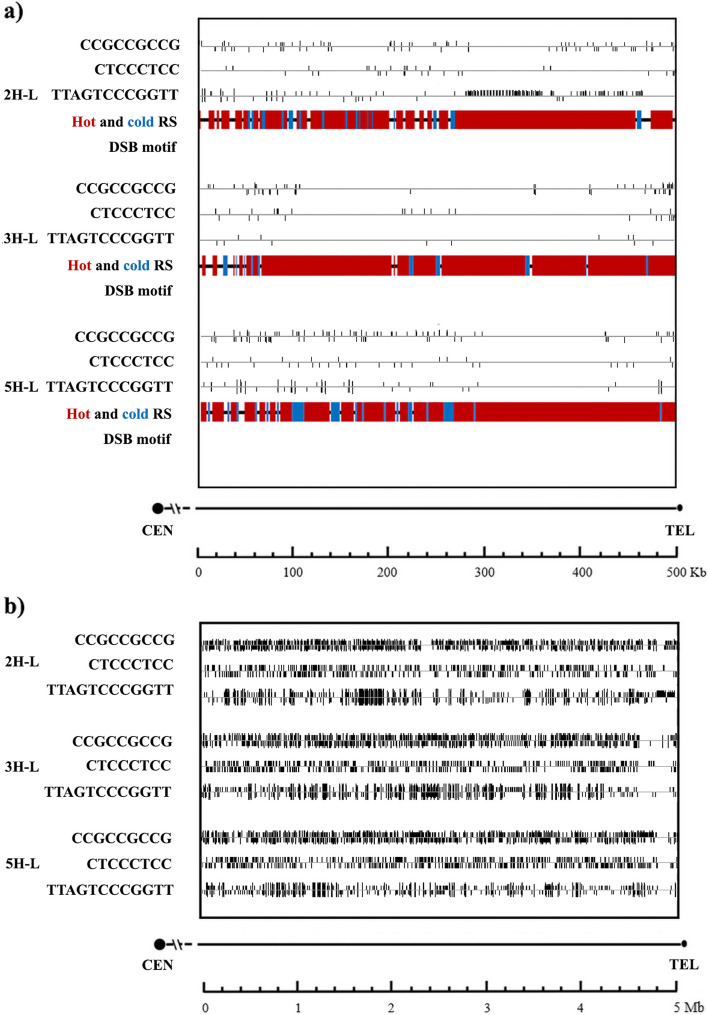
Distribution of predicted hot and cold recombination spots and associated sequences within the distal region of 2H-L, 3H-L and 5H-L barley chromosome arms. **a** Distal subtelomere sequence (500 Kb) of chromosomes were analyzed for the distribution of predicted hot (red) and cold (blue) recombination regions and sequences associated to them. iRSpot-EL was used for prediction of hot and cold recombination spots. Sequences associated with hot recombination spots were identified by MAST (MEME Suite 5.0.5). **b** An extension of the study over the 5 Mb distal subtelomeric region

Distal subtelomere sequence (500 Kb) of long arm chromosomes 2H-L, 3H-L and 5H-L were analyzed for the distribution of predicted hot and cold recombination regions (Fig. 8).

We also examined the distribution of three short sequence motifs (CCGCCGCCG; CTCCCTCC; TTAGTCCCGGTT) that are potentially linked to regions of intense recombination. This analysis covered both the 500 Kb distal subtelomeric region and a broader 5 Mb region (see Fig. 8a, b). Within the 500 Kb distal subtelomere, our analysis revealed a decrease in the occurrence of these motifs in specific regions of the chromosome sequences. Notably, these particular regions align with areas abundant in CG and CpG content, as well as regions enriched in satellite repeats. It is important to mention that a different feature was detected on 2H-L chromosome arm; a high number of TTAGTCCCGGTT motif was found on these zones. We did not see a correlation between the location of these motifs and recombination hot spots. 3H-L chromosome arm presented the lowest number of these motifs, in comparison with the other chromosomes. We also extended our analysis up to 5 Mb of the distal subtelomeric region. A reduction of the number of CCGCCGCCG, CTCCCTCC, TTAGTCCCGGTT motifs was found toward the most distal part of the subtelomere of chromosome arms 3H-L and 5H-L, but we did not see the same reduction of the frequency of CCGCCGCCG motif on 2H-L chromosome arm (Fig. 8 b).

**Fig. 9:**
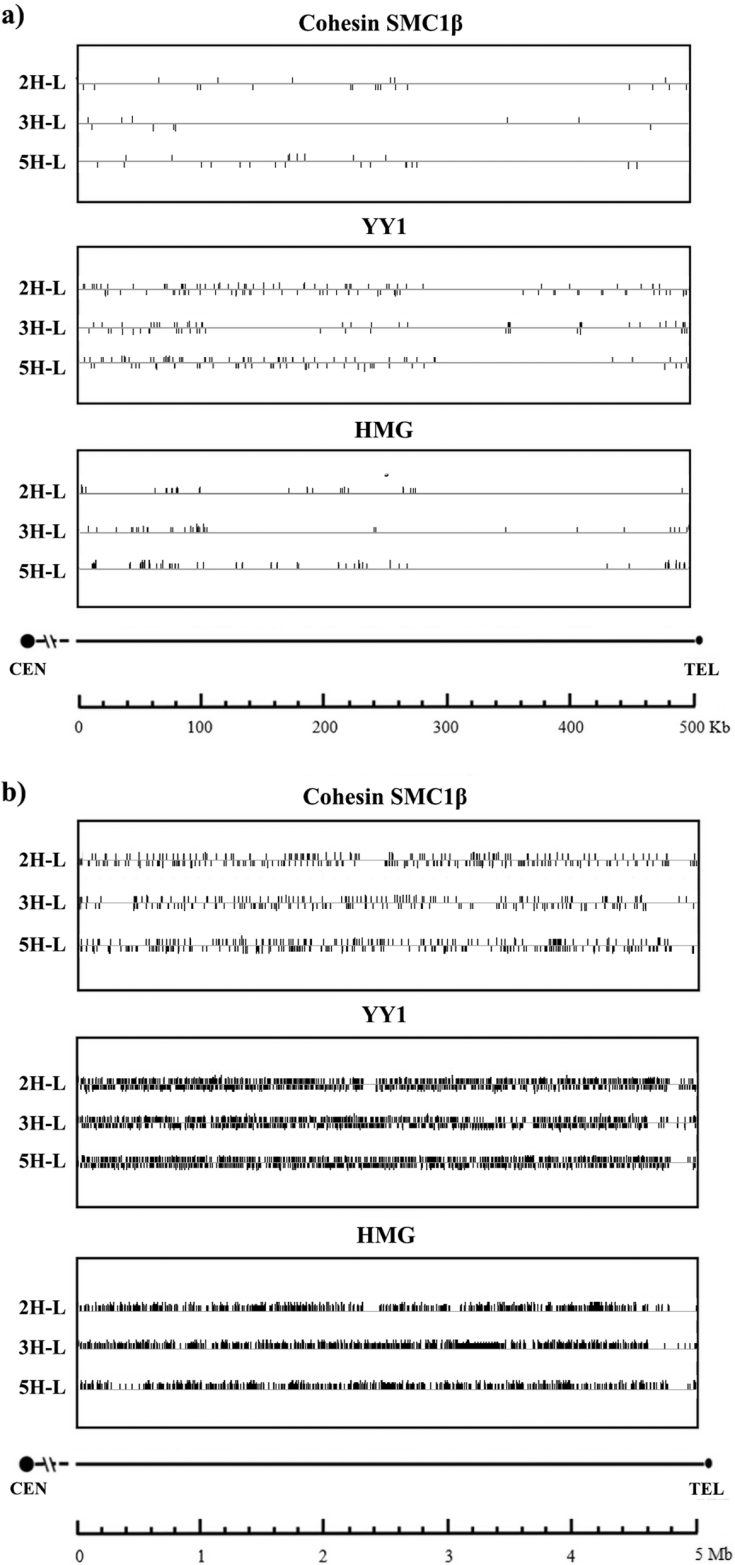
Distribution of predicted binding sites of relevant DNA-binding proteins. **a** Distal subtelomere sequence (500 Kb) of chromosome arms 2H-L, 3H-L and 5H-L were analyzed for the distribution of predicted DNA-binding sites of putative wheat proteins homologous to SMC1β, YY1 and HMG proteins. Sequences were identified and plotted using MAST (MEME Suite 5.0.5). **b** A similar study on the 5 Mb distal subtelomeric region

In this study, we also investigated the distribution of predicted binding sites for pertinent DNA-binding, regulatory, or structural proteins. This analysis was initially conducted within the 500 Kb distal subtelomeric region and subsequently expanded to encompass a 5 Mb region, which included a more proximal subtelomeric section.These proteins were chosen because of their potential implication in chromosome architecture and their putative function in chromosome dynamics such as chromosome approaching and homologous chromosome interactions occurring during early meiosis. DNA binding sites for SMC1β meiosis-specific cohesion protein [81], Ying Yang 1 protein (YY1) [9] and high mobility group proteins HMG [94] were analyzed in the distal subtelomere sequences (500 kb) (Fig. 9). The distribution of potential binding sites for the investigated proteins exhibited specificity unique to each barley chromosome end under examination. This observation lends support to their likely significance as critical factors governing the specificity of chromosome recognition and pairing. We observed that SMC1β, YY1 and HMG proteins biding sites were distributed in the same regions in the chromosome, finding differences in the distribution pattern among 2H-l, 3H-L and 5H-L chromosome arms.

In all the barley chromosome ends we examined, it was evident that regions characterized by a high density of transposable elements and coding genes also exhibited a heightened concentration of binding sites for SMC1β, YY1, and HMG proteins. Conversely, regions with a scarcity of genes and transposable elements displayed a lower density of these binding sites. To further explore this phenomenon, we extended our analysis to encompass a 5 Mb subtelomeric region. Our objective was to investigate whether any variations in the distribution pattern of these binding site proteins could be discerned. Notably, we observed a notable reduction in the density of binding sites in the most distal portion of the subtelomere, near the telomere boundary (Fig. 9b).

## Discussion

The analysis of available barley sequences (NCBI, RefSeq: MorexV3) has shown that most chromosome ends are not sequenced completely or accurately assembled yet, considering that the recent assemblies of most chromosomes do not have the plant terminal telomeric repeat (5´-TTTAGGG-3´, or 3´-AAATCCC-5´ on the complementary strand) [70], as it was observed in bread wheat (polyploid) [1]. Rather than related to ploidy level, miss-assembly must be attributable to the complexity of all chromosome ends, where there are numerous repeats that have not been correctly assembled yet. It is remarkable that telomeric sequences were much longer in barley than in wheat [1]. In plants, telomere length differs depending on the chromosome and the species. These variations imply that telomere length in plants is genetically regulated [24]. Wide variations in telomere length were also seen in differentiating or aging cells in barley (*H. vulgare*) and rice [37, 63].

Our examination of proximal telomere sequences revealed a phenomenon reminiscent of what has been previously documented in rice by Mizuno et al. [64]. This phenomenon involves the addition, deletion, or chromosome-specific substitution of individual nucleotides within the repetitive telomere sequences. Consequently, we chose to commence our analysis by scrutinizing the alterations in the nucleotides constituting these repeating units. While the seven-nucleotide unit typically exhibited conservation, we observed single nucleotide variations throughout the entire telomere sequence. Notably, these variations were more densely concentrated near the junction of the telomere and the chromosome-specific region. All types of mutations, including deletions, additions and substitutions, were found in the three chromosome arms examined.

Across all of these chromosomes, deletions and additions were the predominant types of mutations, while substitutions were relatively rare. Each chromosome exhibited a distinct mutation density and a specific distribution pattern of mutations. In particular, we detected numerous instances of deletions involving one of the "T"s in "TTTAGGG," resulting in a sequence change from "TTTAGGG" to "TTAGGG." Additionally, a lesser number of deletions occurred within "GGG." Conversely, numerous additions of "G" were observed in "GGG," leading to a sequence alteration to "TTTAGGGG." While substitutions were also identified, they were less prevalent compared to deletions and additions in barley chromosome arms 2H-L, 3H-L, and 5H-L. In contrast, in rice, substitutions were discovered to be as prevalent as deletions and additions, as documented by Mizuno et al. [64]. Research on rice further revealed that deletions did not occur randomly,rather, this type of mutation exhibited a bias towards specific bases. Specifically, it was observed that the "T" in "TTT" experienced more deletions than the "G" in "GGG," indicating a preference for thymine mutations [64]. The low-fidelity synthesis of telomere arrays by the telomerase catalytic subunit, which produces 6 bp, or the variety of RNA templates inside telomerases are thought to be responsible for this alteration [88]. Our findings in barley are similar to what was found in rice.

The telomeres of both rice and barley exhibit nucleotide deletions or insertions within the "T" of the canonical "TTTAGGG" repeat. These telomere repeat variations in rice and barley may be attributed to alterations in the genomic sequence responsible for encoding telomerase RNA or changes in its catalytic component, as proposed by Sýkorová et al. [96]. The distribution of these mutations across the entire telomere sequence suggests that a portion of the genomic sequence encoding the RNA template may possess nucleotide deletions, accounting for the deletions observed in barley telomeres, as previously suggested for rice by Mizuno et al. [64]. Having described these frequent variations in telomere repeat sequences, it becomes intriguing to explore their impact on the specificity of interactions with telomeric proteins and their functional consequences. This inquiry holds particular relevance in the context of chromosome recognition and homologous pairing, considering the possibility that homologous chromosomes may initiate their pairing through their telomeres during the bouquet stage of meiosis.Tandem repeats of guanine sequences, denominated G-quadruplexes (G4s), have been analyzed too. G4s, which include four short runs of guanines, are non-canonical structures that can be formed in DNA and RNA sequences when monovalent cations are present. DNA sequences that link the four G-tracts form loops [27]. Technically, a G-quadruplex can be formed by four G-tract DNA sequences that are separated by small runs of non-G bases and each one includes at least three Gs. One consensus sequence of this type of G4s is G_3–5_N_1–7_G_3–5_N_1–7_G_3–5_N_1–7_G_3–5_ (stricter or more flexible models have also been suggested). Replication of DNA, telomere maintenance (they function as capping structures on this regions), and gene transcription are the three most critical processes carried out by G4s [93]. G4s can also bind specific proteins, such as Rif1, POT1-TPP1 complex, HMGB1 protein, TRF2, among others [4, 31, 58, 66, 80]. It is important to remember that proteins have an impact on the composition and operation of G4s [110].

It is worth noting that the genomes of all organisms contain sequences with the potential to form G-quadruplexes, as indicated by Brázda et al. [15]. This widespread presence suggests the potential significance of G-quadruplexes in various biological contexts. In barley, there is a notably higher prevalence of G4 quadruplexes in the telomeric region, in contrast to their frequency in the subtelomeric region and the rest of the chromosome, as detailed in Tables 3 and 4. This disparity may be attributed to the greater abundance of guanine residues comprising the telomeric sequence, as highlighted by Gu et al. [29]. Interestingly, G4s quadruplexes can form superstructures by intramolecular interactions, and more interestingly by intermolecular interactions [75], what could facilitate the direct interaction between chromosomes.

As previously discussed regarding telomere repeat variations, understanding the distribution of G4-quadruplexes along the telomeres, especially in light of variations around the canonical sequence motif, gains significance. Additionally, considering the numerous proteins that bind to telomeres via these G4-quadruplexes, and the potential for direct interactions between DNA molecules from different chromosomes, it becomes intriguing to investigate how these variations might influence the specificity of such interactions and their ensuing functional repercussions. This could be relevant in the context of chromosome recognition and homologue pairing, considering the possibility that homologues could initiate their pairing through their telomeres and distal subtelomeres during the bouquet stage of meiosis.

The subtelomeric region was defined as the stretch between the telomere and the farthest chromosome-specific sequence in previously studied organisms [40, 51, 57]. Before the first active gene at the distal region of subtelomeres, tens of kilobases of highly rearranged and repetitive DNA are a common characteristic thought to be shared by all plants [3, 71, 73, 87, 103, 109]. Nonetheless, with its smaller genome, the model plant *A. thaliana*, paints a different image. *Arabidopsis* subtelomeric regions are short (less than 5 Kb) and relatively simple, but these regions contain short stretches of BAAAA (where B = C, T, or G) and a 32-bp tract composed almost entirely of G [40]. In barley chromosomes, we have not found a sequence pattern that could function as a molecular marker of the limit between telomere and subtelomere or between the distal subtelomere region and the rest of the chromosome. Telomeric sequence and the subtelomere, as well as the distal subtelomere area and the rest of the chromosome, are not clearly delineated in barley by any consistent sequence pattern shared by all chromosomes. Within the 3-Kb ends, we also failed to identify a characteristic that would be common to all three examined chromosomal arms. Aguilar & Prieto [1] could not identify any relevant common sequence motif within these regions in bread wheat chromosomes either. However, we found the short stretches of DNA described in *Arabidopsis* in all three arms analyzed. BAAAA (where B = C, T, or G) was detected in all chromosome arms analyzed in this study and the tract compose of G was found as well in all chromosome arms, but with a smaller number of Gs. This could be related for the fact that barley is a diploid, the same as *Arabidopsis*. In a polyploid organism like bread wheat, these *Arabidopsis* DNA stretches could not be found [1].

For our analysis of the subtelomeric region, we proceeded as it was done in model cereal species like rice and wheat, and in other organisms like humans and *Arabidopsis* [1, 38, 53, 63]. We decided to start our study focusing on the 500 Kb distal region of subtelomeres, but we later expanded the analysis to a larger chromosome region (5 Mb). Subtelomeres are thought to be important for homologue-specific chromosomal pairing and recognition, according to previous studies. For instance, introgressed barley homologous chromosomes failed to detect and associate in pairs when subtelomeres were absent, indicating a crucial role for subtelomeres in these processes [16]. In rye, clustering heterochromatin blocks at the subtelomeres have also been observed, pointing to a potential role of these areas in chromosome connections [61]. In barley, we have focused our analysis on different features of subtelomeric regions that could contribute to pairing specificity. The results of this analysis have been compared with the results obtained in wheat [1].

Concerning the identification of gene sequences within the 500 Kb distal subtelomeric regions of chromosome arms 2H-L-L, 3H and 5H-L, we predicted a total of 43 genes in these three regions (*Ensembl plants*). Unexpectedly, gene density was higher in all chromosome arms when a larger region (5 Mb) was analyzed, unlike what was found in wheat [1, 2]. A relevant feature is the fact that all chromosome arms present a specific pattern of gene distribution. We can see on the 500 Kb analysis that the only common feature is the fact that all three chromosome arms present a higher density of genes at the most proximal part of the subtelomere.

An important feature of TEs was discovered through this analysis: TEs have a chromosome-specific distribution pattern. From a minimum 17% in the 3H-L distal subtelomere and a maximum 54% in the 2H-L, the relative abundance of TEs varies among the chromosomal arms. A similar feature was found in maize and wheat [105, 1, 42, 90]. LTR-type retroelements (Ty1/Copia, Gypsy/DIRS1) are the most prevalent TEs within the distal subtelomeric regions. In general, the most prevalent TEs in plants are LTR-type retroelements [104]. In our study, we found that barley subtelomeres contain an average of approximately 37% TEs, including 33% retroelements and 3% DNA transposons. In a polyploid species like bread wheat, at a genomic scale, TEs represent more than 80% of the whole genome, including 70% retroelements and 13% DNA transposons [18, 46]. Among the retroelements, Gypsy and Copia LTR retroelements are predominant in wheat, while CACTA DNA elements are the most abundant DNA transposons [18]. In barley, we found a higher abundance of Gypsy and Copia LTR retroelements too. Similarities can be observed between these two species, being LTRs the most abundant TEs in both [111]. As suggested by Wicker et al. [106] in barley, TEs are major determinants of overall chromosome structure. The distribution of these TEs within the subtelomeric region could also influence the specificity of the first chromosomal contacts between homologous chromosomes at the start of meiosis.

Repetitive sequences vary in size and complexity among species, and they are more prevalent in species with larger genomes [36]. The subtelomeres of plants like *Arabidopsis*, tobacco, barley, wheat, and potato have a variety of repeat families [10, 32, 39, 54, 100]. In barley, our analysis includes all repeat sequences from satellites to simple repeats (1–5 bp long) and low complexity repeats (poly-purine or poly-pyrimidine stretches, or regions of extremely high AT or GC content). We analyzed these repeats along the 500-Kb distal subtelomeric region of chromosome arms 2H-L, 3H-L and 5H-L. Again, we found that repeat sequence distribution patterns are chromosome-specific, so that chromosomes may be distinguished by this feature in the subtelomeric region. In cereal chromosomes, repeat sequences can be seen as heterochromatic areas. Tandem repeats are relatively prevalent in maize, but they are predominantly found in knob areas and are less prevalent in subtelomeric regions [5, 42]. Rye, barley, and wheat, which are closely related species, exhibit remarkably distinct patterns. A specific characteristic of rye chromosome ends is that they have large heterochromatin blocks [102]. Although the distribution of heterochromatin in wheat and barley chromosomes is complex, their subtelomeres are devoid of significant amounts of heterochromatin [28, 48]. Besides, in wheat, 4AS and 7DS chromosome arms were the only ones that showed a satellite distribution that is similar to the distribution found in barley chromosomes. In all these cases, satellite regions were close to telomeres. It is important to mention that on barley chromosomes we found a higher density of simple repeats in contrast with wheat chromosomes, except for 4AS and 7DS wheat chromosome arms, where the repeats were close to the telomere as well [1].

Genes, transposable elements, and various forms of tandem repeat sequences work together to create a complex and dynamic structure of distal subtelomeres in barley, which appears to be chromosome specific and may contribute to the specificity of chromosome interactions at the start of meiosis. This intricate and dynamic structure of subtelomeres is shared by other plants, including rice with its small genome [102]. Subtelomeres can play a role during the first contacts and pairing of chromosomes, as was previously suggested, and also stabilize the chromosomal ends in the absence of canonical telomeric repeats or protect distal genes from active loss/gain processes within the terminal regions [25, 35, 51].

The functionality of subtelomeres may be determined by simple characteristics like the relative abundance of GC and AT. The whole distal subtelomere sequence (500 Kb) of the long arms of 2H, 3H, and 5H barley chromosome arms differed in GC content and predicted CpG islands. This fact supports the high polymorphism of these subtelomeric regions. It is important to highlight that we found a high density of GC-rich DNA stretches and CpG islands in the most distal part of the subtelomere, near the telomere, in the three chromosome arm ends examined. These solid blocks of GC-rich DNA and CpG islands correspond to areas where we found TREP37 and TREP38 barley satellites. Subtelomeric satellites, near the telomeric region of barley chromosomes, have been shown in previous studies [87]. The presence of genes in animals and plants was shown to be highly associated with GC content and CpG islands [6, 44, 67]. This association is obvious in our work. Most critical, however, is the correlation between GC content and the key processes of recombination and crossover, which mostly occur at the subtelomeric areas and are strongly influenced by the right homologous chromosome interaction earlier in meiosis. Recombination and GC concentration were found to be clearly correlated in maize [95], as in *Triticeae* [22, 30, 68]. Research on *Brachypodium*, maize, and rice found a strong relationship between high GC content, local recombination, and crossover rate [92]. Interestingly, in barley we could also find a good correlation between predicted G4 quadruplexes, GC content and predicted CpG islands, as previously described elsewhere. Apparently, with the species evolution, G4 motifs become more abundant within the promoter regions of genes, especially in genes coding for transcription factors, and there seems to be a negative correlation between methylation state and G4 density [108].

We also analyzed the distribution of short sequence motifs associated with hot recombination regions, previously studied in wheat by Aguilar and Prieto [1]. Hot recombination-related sequences were detected, but we could not find the same correlation between these motifs and hot recombination spots as the correlation found in wheat [1]. In our study and in wheat studies [1] there were obvious disparities among chromosomes in terms of location and size of these areas. Interestingly, an apparent positive correlation was found in barley between frequency of hot recombination spots, transposable elements and satellites, but not with genes.

For all the DNA features analyzed, the subtelomeric areas of the barley chromosome arms investigated here showed a significant degree of polymorphism, which could account for the specificity of the initial chromosome associations in a diploid organism like barley. However, DNA-binding proteins or protein complexes may also be required for the earliest physical interactions of chromosomes to ensure appropriate pairing between homologues [20]. For this reason, we also examined the distribution of DNA-binding proteins that may be involved in chromosome architecture. Candidate proteins were chosen based on their potential involvement in the early meiotic events previously covered in the literature, as well as the presence of known DNA-binding sites. We used putative wheat proteins homologous to human SMC1β meiosis-specific cohesin [81], Ying Yang 1 protein [9] and HMG proteins [94] and looked for their putative DNA-binding sites at the distal subtelomeric sequences on barley chromosomes. Cohesins play a crucial part in sister chromatid cohesion in addition to other meiosis-specific processes such the creation of chromosomal axes, synaptonemal complexes, and reciprocal recombination [20, 33]. Ying Yang 1, an architectural protein, is essential for connecting higher-order chromatin folding in both mammals and *Arabidopsis* [9, 107]. According to previous studies, HMG proteins may interact with AT-rich regions to play a role during the first interactions between homologues before proper pairing [94]. Our observations showed that the distribution of potential binding sites for the proteins under investigation was chromosome-specific, with clear variations in density and distribution among chromosomes. We found an unusual differential distribution of putative binding sites for cohesins, with a very low concentration of these putative sites in comparison with YY1 and HMG binding sites. We found a lower quantity of cohesins, YY1 and HMG binding sites on satellite-rich zones and a higher quantity on gene-rich areas. When comparing the 500 Kb distal region, we could see that binding-site densities varied among chromosomes, but when a larger region of 5 Mb was taken into consideration, these densities were more similar. In barley, a reduction of the number of binding-sites on the most distal part of the subtelomere, near the limit with the telomeric region, can be observed. This reduction is not detectable in wheat studies. In bread wheat, near the telomere region, a higher density of binding-sites was found [1].

The distribution pattern of genes, transposable elements, repeats, GC content, predicted CpG islands, recombination hotspots, G4 quadruplexes, and targeted sequence motifs for key DNA-binding proteins, described in this study, show a high variability both in telomeres and subtelomeres. The molecular basis for the specificity of homologous recognition and pairing in the early chromosomal interactions at the start of meiosis in barley may be provided by these polymorphisms. Our finding of a higher polymorphism in most distal chromosome ends suggest that the most distal part of subtelomeres and telomeres themselves might be particularly relevant for homologue pairing.

## Data Availability

All raw data underlying this article are available in the article.
