# Correction: Telomere and subtelomere high polymorphism might contribute to the specifcity of homologous recognition and pairing during meiosis in barley in the context of breeding

**DOI:** 10.1186/s12864-023-09797-1

**Published:** 2023-11-20

**Authors:** I. M. Serrano-León, P. Prieto, M. Aguilar

**Affiliations:** 1 Plant Breeding Department, Institute for Sustainable Agriculture, Agencia Estatal Consejo Superior de Investigaciones Cient?ficas (CSIC), Avenida Menéndez Pidal S/N., Campus Alameda del Obispo, 14004 Córdoba, Spain; 2 Área de Fisiología Vegetal, Universidad de Córdoba, Campus de Rabanales, Edif. C4, 3? Planta, Córdoba, Spain

**Correction: BMC Genomics 24, 642 (2023)**

10.1186/s12864-023-09738-y

Following publication of the original article [1], it was reported that Table 1 was missing grey highlighting and underlining in the text as described in the table caption. Table 1 has been correctly reproduced in this Correction article, and the original article has been updated.

**Table 1:**
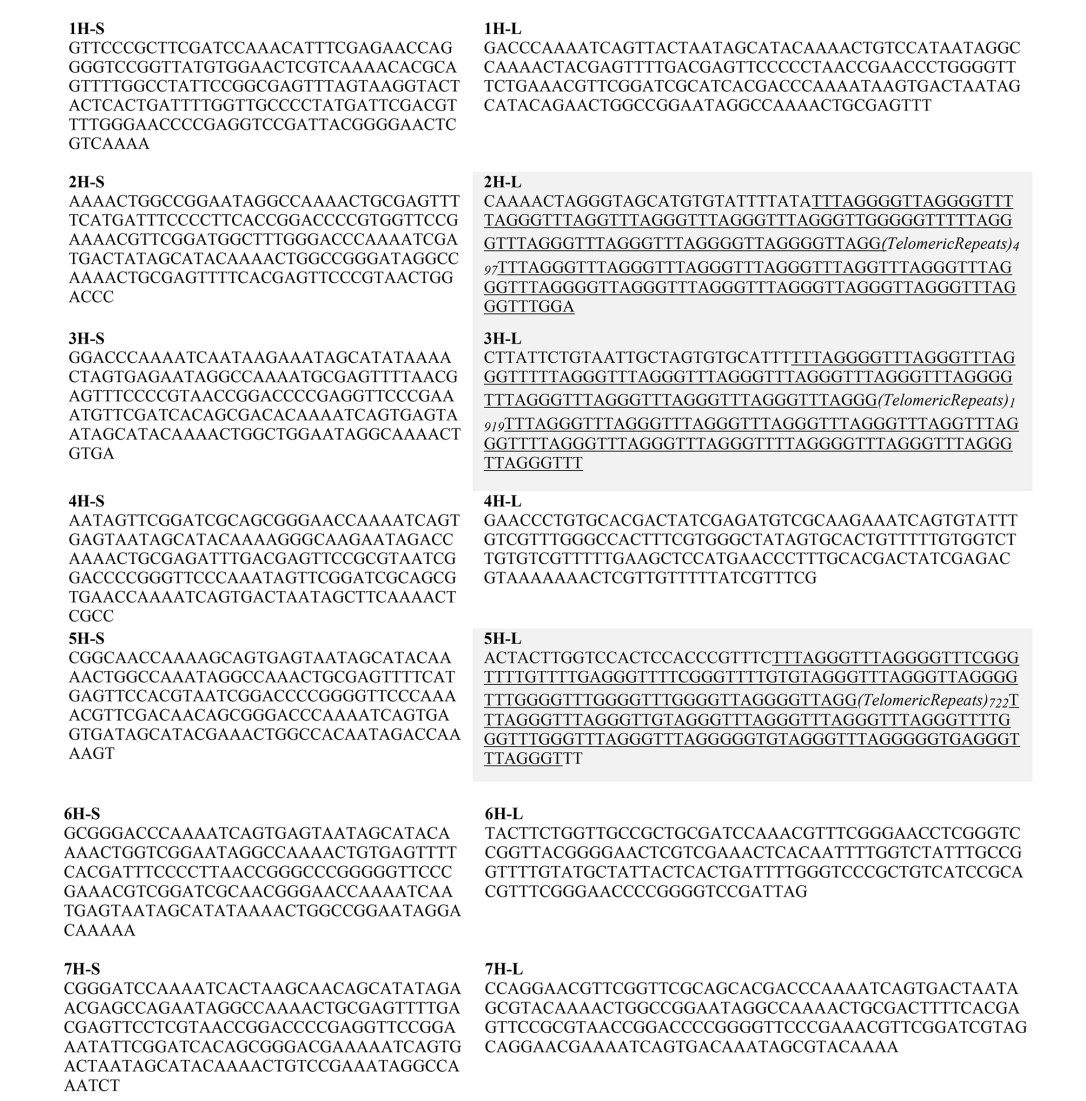
Sequences of barley (*Hordeum vulgare*) chromosome ends. All 14 chromosomes ends are displayed, including both short and long arms of chromosomes. All sequences are presented on the direction of the sequencing, from the end of the short chromosome arm to the end of the long chromosome arm. Chromosome arms that present telomeric repeats are highlighted in grey. The telomeric sequence of 2 H-L, 3 H-L and 5 H-L chromosome arms is underlined. Sequences were obtained by ENA from EBI (RefSeq MorexV3)
